# scGCL: an imputation method for scRNA-seq data based on graph contrastive learning

**DOI:** 10.1093/bioinformatics/btad098

**Published:** 2023-02-24

**Authors:** Zehao Xiong, Jiawei Luo, Wanwan Shi, Ying Liu, Zhongyuan Xu, Bo Wang

**Affiliations:** College of Computer Science and Electronic Engineering, Hunan University, Changsha 410083, China; College of Computer Science and Electronic Engineering, Hunan University, Changsha 410083, China; College of Computer Science and Electronic Engineering, Hunan University, Changsha 410083, China; College of Computer Science and Electronic Engineering, Hunan University, Changsha 410083, China; College of Computer Science and Electronic Engineering, Hunan University, Changsha 410083, China; College of Computer Science and Electronic Engineering, Hunan University, Changsha 410083, China

## Abstract

**Motivation:**

Single-cell RNA-sequencing (scRNA-seq) is widely used to reveal cellular heterogeneity, complex disease mechanisms and cell differentiation processes. Due to high sparsity and complex gene expression patterns, scRNA-seq data present a large number of dropout events, affecting downstream tasks such as cell clustering and pseudo-time analysis. Restoring the expression levels of genes is essential for reducing technical noise and facilitating downstream analysis. However, existing scRNA-seq data imputation methods ignore the topological structure information of scRNA-seq data and cannot comprehensively utilize the relationships between cells.

**Results:**

Here, we propose a single-cell Graph Contrastive Learning method for scRNA-seq data imputation, named scGCL, which integrates graph contrastive learning and Zero-inflated Negative Binomial (ZINB) distribution to estimate dropout values. scGCL summarizes global and local semantic information through contrastive learning and selects positive samples to enhance the representation of target nodes. To capture the global probability distribution, scGCL introduces an autoencoder based on the ZINB distribution, which reconstructs the scRNA-seq data based on the prior distribution. Through extensive experiments, we verify that scGCL outperforms existing state-of-the-art imputation methods in clustering performance and gene imputation on 14 scRNA-seq datasets. Further, we find that scGCL can enhance the expression patterns of specific genes in Alzheimer’s disease datasets.

**Availability and implementation:**

The code and data of scGCL are available on Github: https://github.com/zehaoxiong123/scGCL.

**Supplementary information:**

Supplementary data are available at *Bioinformatics* online.

## 1 Introduction

Single-cell RNA sequencing technology (scRNA-seq) can explain cellular heterogeneity at the single-cell level and discover biological characteristics of different types of cells (Kolodziejczyk *et al.*, 2015). It allows us to reveal relationships between cells with higher resolution and throughput, which is helpful for downstream analyses such as cell clustering (Kiselev *et al.*, 2019), disease treatment (Chowdhury *et al.*, 2021) and inferring cell trajectories (Macosko *et al.*, 2015). However, scRNA-seq data suffer from huge statistical and computational problems, which are very sparse and contain a large number of zero values due to improper sequencing operations or environmental factors (Angerer *et al.*, 2017; Grün *et al.*, 2014).

Facing the problem of the excessive sparseness of scRNA-seq data, researchers have proposed many imputation methods to estimate missing values. Traditional scRNA-seq data imputation methods restore gene expression levels by finding associations between genes. MAGIC (Van Dijk *et al.*, 2018), kNN-smoothing (Wagner et al., 2018) and DrImpute (Gong *et al.*, 2018) estimate the missing value in expression by cell–cell distance. With the extensive application of autoencoders in feature extraction, deepImpute (Arisdakessian *et al.*, 2019), scScope (Deng *et al.*, 2019) and autoImpute (Talwar *et al.*, 2018) learn the latent representation of cells through autoencoders and reconstruct the expression profile of scRNA-seq data from the latent space. Both AutoClass (Li *et al.*, 2022) and scIGANs (Xu *et al.*, 2020) learn data distributions from raw scRNA-seq data and restore gene expression values according to specific cell types. However, these methods rely on the original distribution of scRNA-seq data, and these methods ignore topological structure information of scRNA-seq data.

In recent years, researchers have confirmed that graph neural networks (GNN) can capture the information of adjacent nodes through the graph structure (Zeng *et al.*, 2020). GNN can explore the association between target node and adjacent node, and can effectively enhance the representation of node features. Therefore, GNN methods have been widely used in the analysis of scRNA-seq data. For example, GraphSCI (Rao *et al.*, 2021) estimates missing expression values from scRNA-seq data by reconstructing association graph between genes. scGNN (Wang *et al.*, 2021) builds and aggregates cell-to-cell relationships via a GNN and models heterogeneous gene expression patterns using a left-truncated mixture Gaussian model. scTAG (Yu *et al.*, 2022) aggregates relevant information between adjacent nodes through a GNN and fits cell expression values to the Zero-Inflated Negative Binomial (ZINB) distribution. Although existing GNN-based methods have achieved great success in scRNA-seq imputation, these methods do not consider the global information of the graph, resulting in insufficient cell representation.

Due to cell type labels being limited and difficult to obtain, contrastive learning has shown great potential in self-supervised learning, the core idea behind is to learn better feature representations by maximizing the similarity of positive samples while minimizing the similarity of negative samples. Therefore, exploiting contrastive learning for scRNA-seq data analysis is reasonable. Contrastive-sc (Ciortan and Defrance, 2021) is the first contrastive learning method applied to clustering scRNA-seq data. scNAME (Wan *et al.*, 2022) deeply mines gene correlations and intrinsic cellular structures by introducing a mask estimation task and a contrastive learning framework. Moreover, scNAME ignores the relationships between cells. With the rise of graph contrastive learning in the field of graph representation learning (Thakoor *et al.*, 2021), it will become a new idea to use graph contrastive learning to capture the relationship between cells and recover missing gene expression values.

**Fig. 1.**
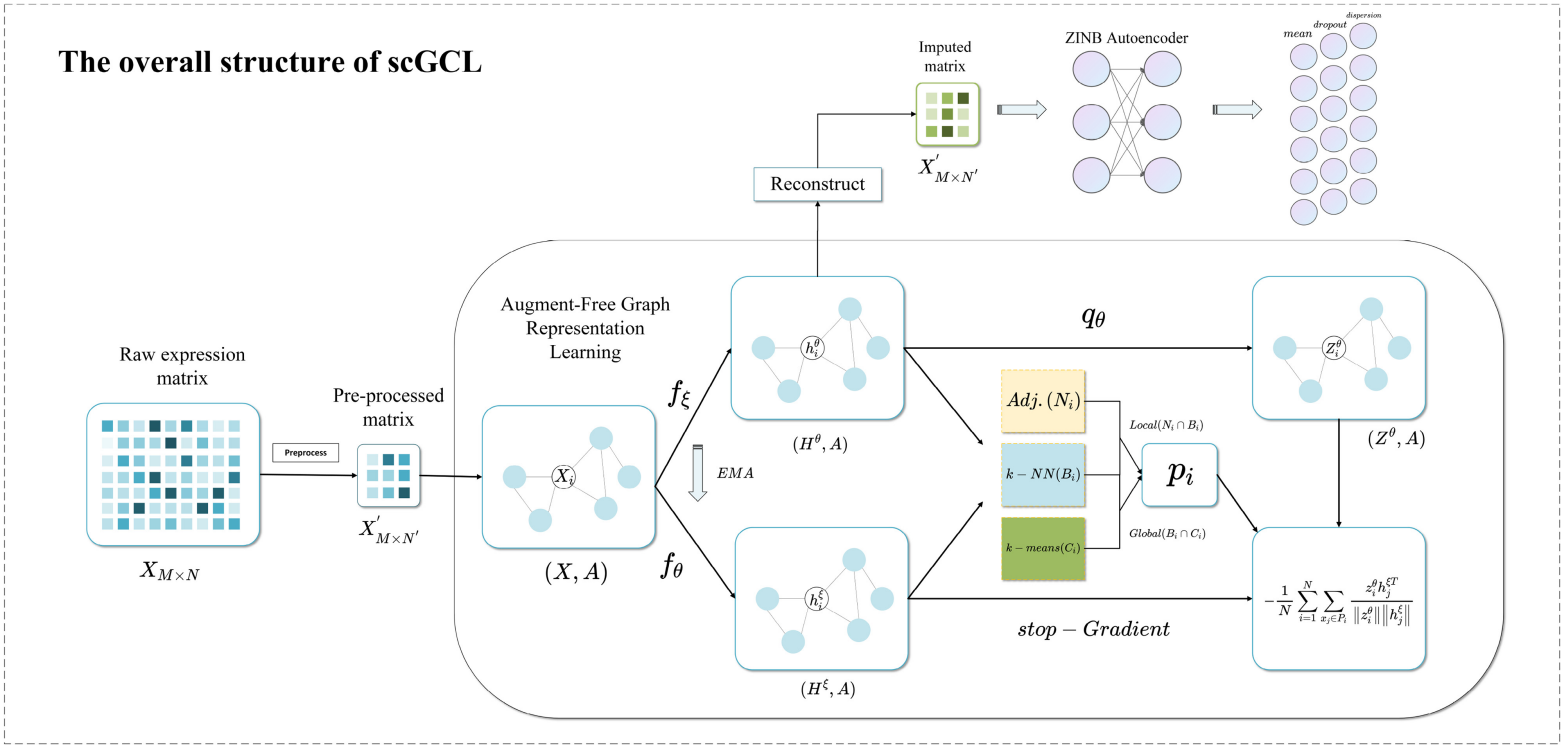
The architecture of scGCL. scGCL takes the gene expression matrix produced by scRNA-seq as input. scGCL consists of two parts: AFGRL framework and ZINB autoencoder. AFGRL is a graph contrastive learning framework, which can transform the input gene expression data into two different views through graph convolution operation and use the node embedding generated by the target encoder $f_{\xi }$ as the final reconstructed gene expression matrix. To capture the global probability distribution of scRNA-seq data, scGCL reconstructs the gene expression matrix through the zero-inflated negative binomial (ZINB) autoencoder

Therefore, this article proposes a scRNA-seq data imputation method based on Graph Contrastive Learning (scGCL). The architecture of scGCL is shown in Figure 1. First, we use the pre-processed scRNA-seq data expression profile to construct the cell graph through the KNN algorithm. Then, scGCL generates two similar representations through graph convolution with the online encoder and the target encoder. To comprehensively capture the topological information of the cell graph, we exploit a graph contrastive learning framework to enhance the cell representation. Finally, we apply the ZINB autoencoder to reconstruct the expression profile of the scRNA-seq data based on online representations, which effectively capture the global probabilistic information of the data. scGCL achieves promising performance on three downstream tasks of cell clustering, gene imputation and cell development trajectory inference, compared to three existing imputation methods and one state-of-art clustering tool. To further investigate the performance of scGCL on the complex disease, we apply it to the Alzheimer’s disease (AD) dataset to elucidate its role in restoring gene expression and enhancing the selection pattern of differentially expressed genes (DEG).

## 2 Materials and methods

### 2.1 Data pre-processing

The expression matrix *X* of the scRNA-seq data is taken as the input raw data, where $X_{i,j}$ represents the raw expression value of the *j*th gene (1≤j≤O) on the *i*th cell (1≤i≤N). To reduce noises in the scRNA-seq data, we pre-process the raw gene expression profiles by the following pre-processing methods. First, data filtering and quality control are the first steps in scRNA-seq data pre-processing. Therefore, we refer to scGNN (Wang *et al.*, 2021), keeping only genes with non-zero expression in more than 1% of cells and cells with non-zero expression in more than 1% of genes. Next, since the data in the count matrix are discrete and affected by the size factor, we normalize it by the size factor then transform discrete values through the log function. The normalization function is defined as:


(1) $$\begin{array}{c} N(X_{i,j})=ln(m(X)\frac{X_{i,j}}{\sum_{o}X_{i,o}}), \end{array}$$

where m(X) represents the median of the total cell expression counts. Finally, we select the top *t* highly variable genes based on the normalized discrete values computed by the *scanpy* package (Wolf *et al.*, 2018). Generally, we select 2048 highly variable genes for training and use a consistent pre-processing method before running all baseline methods.

### 2.2 Cell graph

Similar to previous work (Yu *et al.*, 2022), we use the KNN algorithm to generate the cell graph *A*, and each node in the graph represents a cell. And we assign an edge between cell a and cell b if cell b is one of the k nearest nodes to cell a, where k is set to 15 in this article. Due to the high sparsity characteristic of scRNA-seq data, we apply cosine similarity to describe the distance between cells. In this article, we use graph convolution to generate two similar views and preserve the information of adjacent nodes and associated nodes in the graph *A* through these two views.

### 2.3 Graph contrastive learning framework

In order to better capture the topological information of graphs, we introduce a graph contrastive learning framework using Augment-free Graph Representation Learning (AFGRL) (Lee *et al.*, 2021) as the main structure. AFGRL can select appropriate samples for enhancement while considering global semantics and local information without changing the topology of the graph. For each cell $x_{i}\in X$, we generate two different node representations of the cell based on the online encoder $f_{\theta }(*)$ and the target encoder $f_{\xi }(*)$, which are considered as positive sample pairs. In scGCL, both the online encoder and the target encoder use a graph convolution structure, which takes the original cell graph *A* and the cell gene feature *X* as input, $H^{\theta }=f_{\theta }(X,A)$ and $H^{\xi }=f_{\xi }(X,A)$ are two different node representations of the same cell, respectively. Then, for each cell $x_{i}\in X$, we compute the cosine similarity among it and the rest of the cells, used to filter nodes with different semantic information. The cosine similarity is calculated as follows:


(2) $$\begin{array}{c} \mathrm{sim}(x_{i},x_{j})=\frac{h_{i}^{\theta }\cdot h_{j}^{\xi }}{\left\|h_{i}^{\theta }\right\|\left\|h_{j}^{\xi }\right\|},\forall x_{i}\in X \end{array},$$

where $h_{i}^{\theta }$ is the online embedding of cell $x_{i}$, $h_{j}^{\xi }$ is the target embedding of the remaining cells $x_{j}$, and $\mathrm{sim}(x_{i},x_{j})$ is the similarity information between two different embeddings. The similarity information is taken as the distance between two cell embeddings, we find all cell nodes similar to the cell $x_{i}$ through the k-nearest neighbor algorithm and mark them as the set $B_{i}$. It is worth noting that the hyperparameter $k_{b}$ used by the KNN algorithm used to construct $B_{i}$ is different from the *k* used to construct the cell graph and the default $k_{b}=4$. The nodes in the set $B_{i}$ are used as scGCL candidate positive nodes.

To efficiently select real positives nodes from the set $B_{i}$, we explore distinct cell graph information from the perspective of local structural and global semantics. First, cells associated with $x_{i}$ in the adjacency matrix tend to share the same label as $x_{i}$ due to the smoothness assumption (Zhu *et al.*, 2003). We denote the set of nodes associated with the target node on the adjacency matrix *A* as $N_{i}$. Therefore, we compute the intersection between the neighboring node $N_{i}$ and the nearest neighbor $B_{i}$, which is considered as the local positives of the cell $x_{i}$, i.e. $B_{i}\cap N_{i}$. Next, to discover non-adjacent nodes that share global semantic information with the query node, we apply the K-means algorithm on the target embedding $h_{j}^{\xi }$ to the cluster, where we consider the set of nodes that belong to the same cluster as the target node, i.e. $C_{i}$. $C_{i}$ is named the global semantically similar node of the target node. The intersection of the nearest neighbor node $B_{i}$ and the global semantically similar node $C_{i}$ is used as global positives, i.e. $B_{i}\cap C_{i}$. Finally, we consider the set of all real positives $P_{i}$ that are ultimately used for node augmentation as follows:


(3) $$\begin{array}{c} P_{i}=(B_{i}\cap N_{i})\cup (B_{i}\cap C_{i}) \end{array}.$$

scGCL enhances the representation of the original cell node $x_{i}$ by minimizing the cosine distance. The objective function is as follows:


(4) $$\begin{array}{c} z_{i}^{\theta }=q_{\theta }(f_{\theta }(x_{i},A)),\forall x_{i}\in X \end{array}$$

(5) $$\begin{array}{c} L_{\theta ,\xi }=-\frac{1}{N}\sum_{i=1}^{N}\sum_{x_{j}\in P_{i}}\frac{z_{i}^{\theta }f_{\xi }{\left(x_{j},A\right)}^{T}}{\left\|z_{i}^{\theta }\right\|\left\|f_{\xi }(x_{j},A)\right\|} \end{array},$$

where $z_{i}^{\theta }$ represents the prediction of the online embedding $h_{i}^{\theta }$ through the predictor network $q_{\theta }(\cdot )$. *A* represents the cell graph. During the imputation process of scGCL, the online embedding $H^{\theta }$ is used for the reconstruction of the scRNA-seq expression profile.

### 2.4 Autoencoder based on ZINB distribution

In order to capture the characteristics of scRNA-seq data such as high sparsity and variance greater than the mean, we introduced the ZINB model-based autoencoder as in scTAG (Yu *et al.*, 2022) to reconstruct the expression profile of scRNA-seq data. In general, the ZINB model consists of three important parameters: the dispersion degree (θ) and mean (μ) of the negative binomial distribution, and an additional coefficient (π) representing the dropout rate. The ZINB model-based autoencoder completes the reconstruction of scRNA-seq data by estimating θ, μ and π. The ZINB model-based autoencoder reconstruct scRNA-seq expression profiles is defined as follows:


(6) $$\begin{array}{c} NB(x_{i}|\mu ,\theta )=\frac{\Gamma (x_{i}+\theta )}{x_{i}!\Gamma (\theta )}{\left(\frac{\theta }{\theta +\mu }\right)}^{\theta }{\left(\frac{\mu }{\theta +\mu }\right)}^{x_{i}} \end{array},$$

(7) $$\begin{array}{c} \mathrm{ZINB}(x_{i}|\pi ,\mu ,\theta )=\pi \delta_{0}(x_{i})+(1-\pi )NB(x_{i}) \end{array},$$

where μ and θ represent the mean and variance, respectively; π represents the probability that the zero value exists in the scRNA-seq data. To introduce the ZINB distribution model into scGCL, we apply three fully connected layers to estimate the important parameters π,μ,θ in the ZINB distribution:


(8) $$\begin{array}{c} \pi =\mathrm{sigmoid}(W_{\pi }f_{D}(Z)) \end{array},$$

(9) $$\begin{array}{c} \mu =\text{exp}(W_{\mu }f_{D}(Z)) \end{array},$$

(10) $$\begin{array}{c} \theta =\text{exp}(W_{\theta }f_{D}(Z)) \end{array},$$

where $f_{D}$ is a fully connected neural network with two hidden, the number of nodes in the hidden layer is 512 and 256, which are consistent with scTAG. *W* represents the updated weights in the neural network; π, μ and θ are used to simulate ZINB. The three parameters in the distribution represent dropout probability, mean and variance, respectively. ZINB model-based autoencoder utilizes the negative log-likelihood of ZINB as the reconstruction loss for the original data $x_{i}$, which is defined as follows:


(11) $$\begin{array}{c} L_{\mathrm{ZINB}}=-\log(\mathrm{ZINB}(x_{i}|\pi ,\mu ,\theta )) \end{array}$$

### 2.5 Update strategy for scGCL

scGCL is a graph self-supervised method based on contrastive learning. First, scGCL learns the representation of the target node by reducing the similarity between two node representations. The objective function is defined as $L_{\theta ,\xi }=\left\|q_{\theta }(g_{\theta }(h_{1}))-g_{\xi }(h_{2})\right\|$, where $g_{\theta }$ and $g_{\xi }$ represent two projectors. Based on the contrastive learning method, $x_{2}$ is put into the online encoder and $x_{1}$ is put into the target encoder to get a symmetric loss ${\tilde{L}}_{\theta ,\xi }$. The final contrast loss is $L_{\theta ,\xi }^{\mathrm{Contrast}}=L_{\theta ,\xi }+{\tilde{L}}_{\theta ,\xi }$. Next, to prevent the collapse problem, we introduce an exponential moving average method in the training process of the two-view encoder, which delays the update of the weights of the target encoder (Chen and He, 2021). Finally, the optimization function of scGCL is as follows:


(12) $$\begin{array}{c} L=\gamma_{1}L_{\mathrm{Contrast}}+\gamma_{2}L_{\mathrm{ZINB}}+\gamma_{3}L_{\mathrm{Reconstruct}} \end{array},$$

where $L_{\mathrm{Contrast}}$, $L_{\mathrm{ZINB}}$ and $L_{\mathrm{Reconstruct}}$ represent the contrast loss, ZINB loss and reconstruction loss of scRNA-seq expression profiles, respectively. $\gamma_{1}$, $\gamma_{2}$ and $\gamma_{3}$ are hyperparameters used to control the loss. In the experiment, $\gamma_{1},\gamma_{2},\gamma_{3}$ are set to 1.5,1.0,1.0.

## 3 Results

### 3.1 Experimental settings

scGCL uses the KNN algorithm to construct the cell graph and the similar cell set $B_{i}$ respectively, we set k=15, distances measures=cosine, and $k_{b}=4$ as the default parameters. During the training of scGCL, we use the Adam optimizer for optimization, where learning_rate is set to 0.001, and *epoches* is set to 300. For all baseline methods, hyperparameters are set to be consistent with the original paper. All our experiments are on a Ubuntu server with NVIDIA RTX 3090Ti GPU and 24 GB memory.

We evaluate the performance of scGCL on 14 scRNA-seq datasets from different tissues, species and sequencing platforms. The details of the 14 scRNA-seq datasets used in the experiments are described in Table 1. In particular, the number of cells ranges from 870 to 18 664, and the number of genes ranges from 10 850 to 33 658.

**Table 1.** Summary of the scRNA-seq datasets

Dataset	Cell	Gene	Cell type	Platform	Reference
Adam	3660	23 797	8	Drop-seq	Adam *et al.* (2017)
Alzheimer	13 215	10 850	8	10x	Grubman *et al.* (2019)
Muraro	2122	19 046	9	CEL-seq2	Muraro *et al.* (2016)
Plasschaert	6977	28 205	8	inDrop	Plasschaert *et al.* (2018)
Qx_Bladder	2500	23 341	4	10x	Schaum *et al.* (2018)
Qx_Limb_Muscle	3909	23 341	6	10x	Schaum *et al.* (2018)
Qx_Spleen	9552	23 341	5	10x	Schaum *et al.* (2018)
QS_Diaphragm	870	23 341	5	Smart-seq2	Schaum *et al.* (2018)
QS_Limb_Muscle	1090	23 341	6	Smart-seq2	Schaum *et al.* (2018)
QS_Lung	1676	23 341	6	Smart-seq2	Schaum *et al.* (2018)
Romanov	2881	21 143	7	Smart-seq2	Vento-Tormo *et al.* (2018)
Tosches_turtle	18 664	23 500	15	Drop-seq	Tosches *et al.* (2018)
Wang_lung	9519	14 561	2	10x	Wang *et al.* (2018)
Young	5685	33 658	11	10x	Young *et al.* (2018)

### 3.2 Gene expression recovery and clustering analysis

**Fig. 2.**
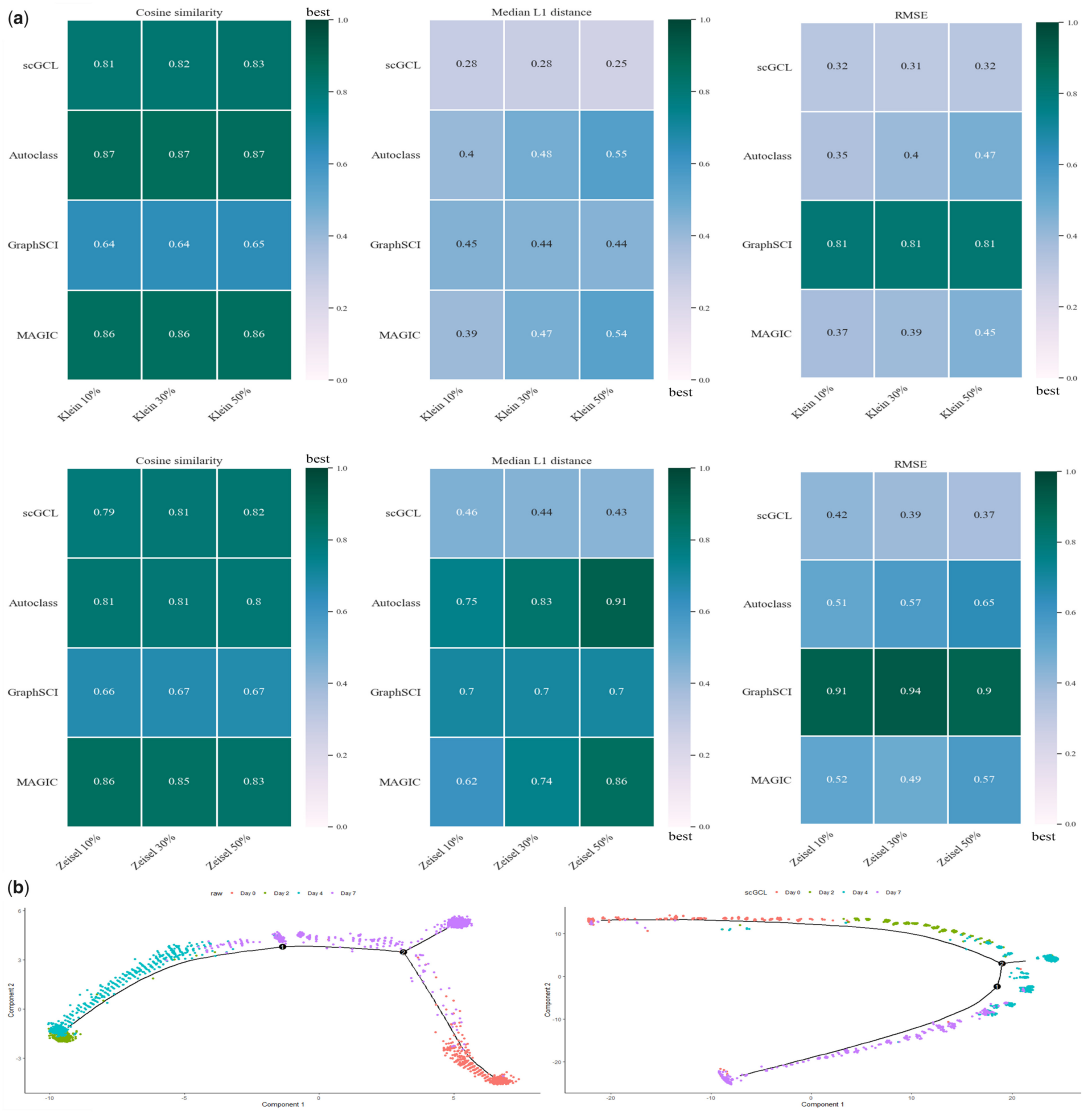
Imputation performance comparison. (**a**) Comparison of cosine similarity and median L1 distance among scGCL and other three imputation tools at 10%, 30% and 50% synthetic dropout rates. The median L1 distance and RMSE rank best from the lower to the upper value and the cosine similarity ranks best from the higher to the lower value. (**b**) Pseudotime analysis using the raw expression matrix and scGCL imputed matrix of the Klein dataset via Monocle

To assess the imputation performance of scGCL, we conduct experiments using two gold-standard cell-annotated datasets [Zeisel (Zeisel *et al.*, 2015) and Klein (Klein *et al.*, 2015)] as the benchmarks. According to scVI (Lopez *et al.*, 2018) strategy, we simulate the dropout phenomenon by randomly changing a certain number of non-zero expression values to zero (10%, 30% and 50%). The median L1 distance, RMSE and cosine similarity between the original data and the imputed values are used as criteria for the ability to restore gene expression levels. The higher the correlation and the lower the median L1 distance and RMSE, the better the performance. These criteria are calculated to compare scGCL with AutoClass, GraphSCI and MAGIC. The cosine similarity score of scGCL is competitive with AutoClass and MAGIC, while GraphSCI performs the worst. It is worth noting that the Median L1 distance score and RMSE score of scGCL ranks at the best for 10%, 30% and 50% synthetic dropout rates in all datasets (Fig. 2a).

Pseudotime analysis of scRNA-seq data is an important downstream analysis, which can reveal developmental patterns of cells. Dropout events affect the trajectory reconstruction of scRNA-seq data, and imputation methods can effectively alleviate this phenomenon (Dai *et al.*, 2022). We perform four imputation methods on the raw data from Klein dataset and use the Monocle (Qiu *et al.*, 2017) to infer the trajectory of cell development. Only scGCL restores complex structures not exhibited in the raw data, demonstrating a well-aligned trajectory path of cell development (Fig. 2b). In particular, compared to GraphSCI, scGCL separates Day 2 and Day 4 cells well, and exhibits a better developmental trajectory (Supplementary Fig. S1).

Clustering analysis of scRNA-seq data is an essential analysis task, which affects the discrimination of cell types and subtypes. To evaluate the Clustering analysis of scGCL, we compare it with four state-of-the-art baseline methods on 14 datasets. Among these four baseline methods, GraphSCI and scTAG are based on graph convolution, AutoClass is based on pre-clustering, and MAGIC is the traditional scRNA-seq imputation method. Two traditional Clustering analysis criteria, adjusted rand index (ARI) (Rand, 1971) and normalized mutual information (NMI) (Strehl and Ghosh, 2002) are used to evaluate the Clustering performance of scGCL. When using baseline methods, we choose the same pre-processing method to ensure the fairness of the clustering results. On the Quake_Smart-seq2_Diaphragm dataset, the scTAG loss cannot be calculated during the pre-training process, so the pre-trained epoches are modified to 500.

**Fig. 3.**
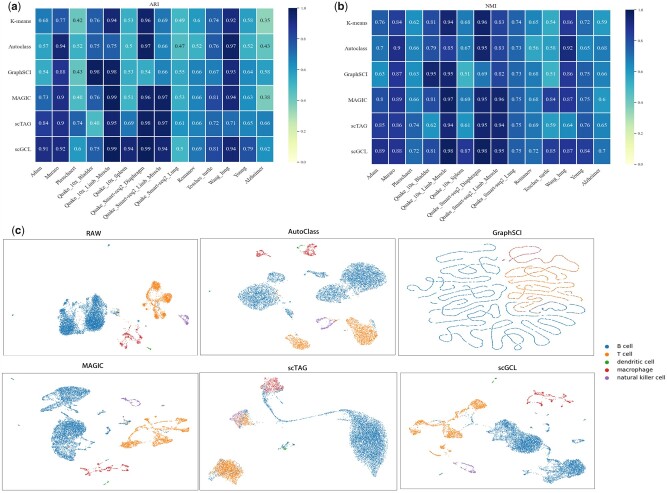
Clustering results of scGCL. (**a**) Visualization of ARI metrics for multiple different alignment methods on 14 scRNA-seq datasets. The ARI ranks best from the higher to the lower value. (**b**) Visualization of NMI metrics for multiple different alignment methods on 14 scRNA-seq datasets. The NMI ranks best from the higher to the lower value. (**c**) UMAP plot for raw and imputed data on Qx_Spleen

**Fig. 4.**
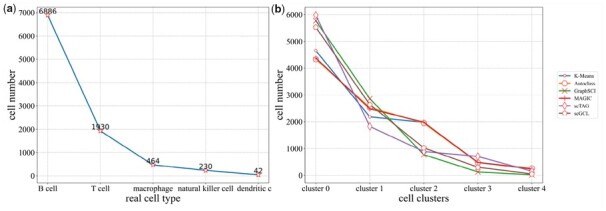
Identify the number of cells in different cell clusters of Qx_Spleen. (**a**) The real cell number of Qx_Spleen on different cell types. (**b**) By different methods, identify the number of cells of different cell clusters on Qx_Spleen

For 14 scRNA-seq datasets, the Clustering results of scGCL slightly outperforms on the average all baseline methods on most datasets (Fig. 3a and b). The average ARI and NMI of scGCL across all 14 datasets are 0.82 and 0.80 with the second best values of 0.80 and 0.75 (Supplementary Fig. S2). In particular, on the Quake_10x_Spleen dataset, the Clustering results of scGCL far outperform other comparison methods, with ARI and NMI reaching 0.94 and 0.87. At the same time, we find that state-of-the-art graph convolutional embedding methods have certain drawbacks through UMAP visualization. scTAG measures the similarity between cells and cluster centers, making cells with ambiguous clusters move closer to the wrong cell clusters. For example, on the Quake_10x_Spleen dataset, a subset of macrophage cells, B cells, and T cells are clustered together. GraphSCI applies association information between genes, which destroys the original properties of scRNA-seq data. Therefore, although GraphSCI outperforms the raw data in terms of clustering effect, its UMAP visualization is affected by the gene graph, and it is difficult to show the original biological characteristics. scGCL not only effectively distinguishes different types of cells but preserves the biological properties of scRNA-seq data (Fig. 3c). On the Quake_10x_Spleen dataset, we observe the number distribution of five different cell types from high to low cell numbers and report the number of cells present in each cluster detected by different methods (Fig. 4a and b). The number of cells detected by scGCL on different cell clusters is the closest to the number of cells of the real cell type, which indicates that scGCL maintains the characteristics of the cell type. In summary, scGCL can effectively eliminate the dropout events of scRNA-seq data and facilitate downstream analysis.

### 3.3 Hyperparameter analysis

**Fig. 5.**
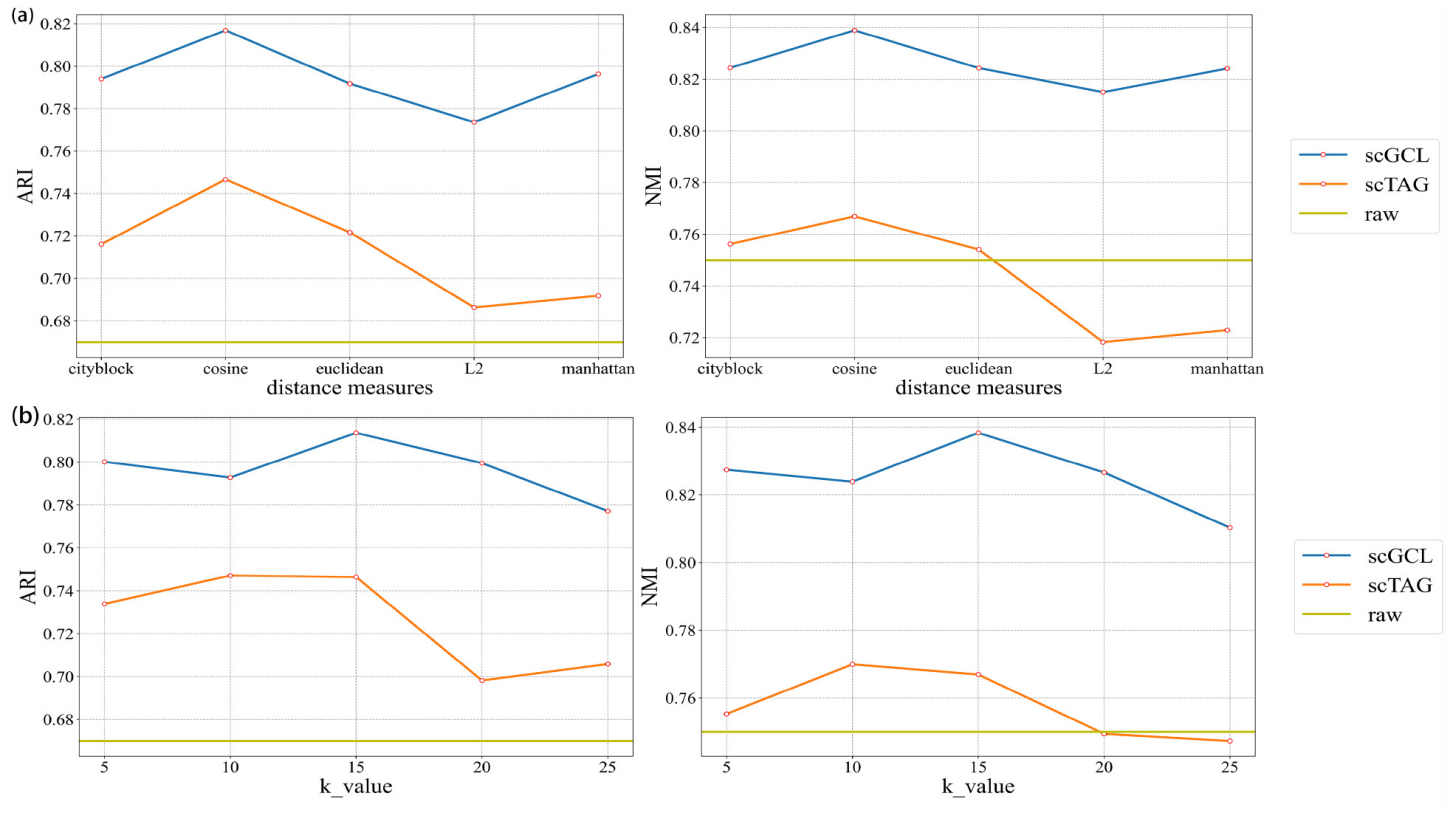
Parameter analysis. (**a**) Comparison of the NMI and ARI values with different neighbor parameters, *k*. (**b**) Comparison of the NMI and ARI values with different distance parameters

The *k* and the distances measures are important hyperparameters for constructing the cell graph and determining the cell–cell relationship in the graph. Since among all baselines, only scTAG uses the K-NN method to construct cell graphs like scGCL, we choose it as the only comparison method for hyperparameter experiments. To investigate the effect of *k* and distance measures in constructing the cell graph on scGCL, we experiment on all datasets across various *k* and distance measures. In the experiment, we set *k* to be 5, 10, 15, 20 and 25, respectively, and set the distances measures to be ‘city-block’, ‘cosine’, ‘euclidean’, ‘L2’ and ‘manhattan’, respectively. Figure 5a and b shows the average ARI and NMI values of all datasets under different k values and distance measures. The experimental results of all datasets are reported in Supplementary Tables S1–S4. From Figure 5a, it can be observed that using the ‘cosine’ distance to frame the cells can capture the distance information between cells more effectively. This phenomenon may be caused by the high sparsity of scRNA-seq data, and the cosine distance can better characterize the relationship between cells. From Figure 5b, we can observe that scGCL and scTAG achieve the best clustering effect when k=15, and scGCL is always better than scTAG. Therefore, we set the parameter k=15 and set the distance to the ‘cosine’ distance.

### 3.4 Ablation study

To evaluate the impact of different components of scGCL on the imputation results, we ablate different components on Adam and Romanov datasets. In particular, we specify two different cases: (i) No ZINB-based encoder and retain the AFGRL and graph convolutional models. (ii) Only the ZINB-based encoder is kept, and the graph convolution and AFGRL models are removed. As shown in Table 2, the ZINB distribution is a necessary part of the method, which proves the effect of capturing the global probability distribution of scRNA-seq data. Furthermore, we observe that the clustering results of Only_ZINB are better than those of scGCL on the Romanov dataset, implying that graph representation learning sometimes disrupts the probabilistic features of scRNA-seq data. Therefore, the balance of graph representation learning and ZINB distribution is a critical training objective during training.

**Table 2.** Ablation study measured by NMI and ARI values

Methods	Metrics	raw_Kmeans	Only_AFGRL	Only_ZINB	scGCL
Adam	ARI	0.6762	0.8159	0.8357	0.9067
	NMI	0.7552	0.8364	0.8596	0.8927
Romanov	ARI	0.5966	0.6777	0.7981	0.6944
	NMI	0.6547	0.7022	0.7556	0.7159

### 3.5 Case study: Alzheimer’s disease

We apply scGCL to the public Alzheimer’s disease (AD) dataset and keep all hyperparameters constant. From Figure 3a and b, we observe that the clustering indicators ARI and NMI of scGCL on the AD dataset reached 0.62 and 0.70, respectively. As can be observed in Supplementary Figure S3a, scGCL separates AD and control cells more efficiently than the raw scRNA-seq data. As shown in Supplementary Figure S5, GraphSCI confuses AD cells and control cells and can no longer discriminate them effectively. Next, we use the state-of-the-art *wilcoxon* method in the *scanpy* package to find differentially expressed genes (DEGs) on the raw scRNA-seq data, the GraphSCI-imputed scRNA-seq data, and the scGCL-imputed scRNA-seq data. Among all the DEGs, we identify 20 DEGs related to their types in different cell types.

As we can observe from Supplementary Figures S6–S8, scGCL most significantly enhances the expression levels of differential genes. Further, we use these DEGs to perform pathway enrichment analysis on the KEGG database. scGCL can most effectively discover potential disease pathways (Supplementary Figs S3b, S4b and S5b), such as Alzheimer’s disease and Huntington’s disease. scGCL is able to effectively account for the missing true expression values in the original dataset, and the pattern of DEG became clearer. In particular, GraphSCI confounds the differences between AD cells and control cells, resulting in disrupted differential gene expression patterns and ineffective discovery of the correct disease pathways. Finally, we select 20 marker genes such as ‘CPSF3L’ and ‘ACAP3’, which are confirmed to impact cell classification substantially. scGCL maintains the original expression pattern of specific genes while making the DEG pattern clearer (Supplementary Figs S9–S12). Therefore, scGCL can effectively analyze disease-related datasets and provide a reference for differential gene expression analysis.

## 4 Discussion

scRNA-seq analysis still faces fundamental challenges, including high sparsity of expression profiles and complex differential gene expression patterns. Imputation of scRNA-seq data can effectively restore gene expression levels and facilitate downstream analysis. In this article, we propose an imputation method for scRNA-seq data scGCL based on graph contrastive learning. First, we construct a cell graph from the relationships between cells. Next, we apply a graph contrastive learning framework to scRNA-seq data, which can capture both global semantic and local information to better enhance the representation of nodes in the graph. Finally, we embed the ZINB-based autoencoder into the scGCL framework, which can effectively capture the global probability distribution of scRNA-seq data and reconstruct the data.

To validate the performance of scGCL on scRNA-seq data imputation, we evaluate scGCL and other state-of-the-art baseline methods on multiple downstream analysis tasks such as clustering performance, recovering gene expression levels and pseudo-time analysis. scGCL demonstrates its effectiveness on scRNA-seq data imputation through extensive experiments. Furthermore, we verify the influence of different hyperparameters on the clustering results of scGCL, and the importance of each part on scGCL through ablation experiments. In the end, we perform a case study on the Alzheimer’s disease dataset, validating that scGCL enables clearer gene expression patterns from scRNA-seq data. In the future, we will continue to enhance the balance of scGCL and apply it to the integration of single-cell multi-omics data. In addition, we hope to provide stronger interpretability for the model by integrating the topic model.

## Supplementary Material

btad098_Supplementary_Data
