# Supplementary material for: scGCL: an imputation method for scRNA-seq data based on graph contrastive learning
Source: Bioinformatics. 2023 Feb 24;39(3):btad098. doi: 10.1093/bioinformatics/btad098 (PMC9991516; doi:10.1093/bioinformatics/btad098)
Supplement: btad098_Supplementary_Data [file btad098_supplementary_data.zip › SupplementaryMaterials_S2_tab.docx]

**Table S1. Imputation results by scGCL with different distance measures**

| **Distance measures** |  | **cityblock** | **cosine** | **euclidean** | **L2** | **manhattan** |
| --- | --- | --- | --- | --- | --- | --- |
| Adam | ARI | 0.8643 | **0.9089** | 0.8979 | 0.8966 | 0.8619 |
|  | NMI | 0.8633 | **0.8943** | 0.8863 | 0.8859 | 0.8624 |
| Alzheimer | ARI | 0.4992 | **0.6197** | 0.6095 | 0.5222 | 0.5823 |
|  | NMI | 0.6436 | **0.7004** | 0.6845 | 0.6517 | 0.6698 |
| Muraro | ARI | 0.8432 | **0.9152** | 0.8850 | 0.9051 | 0.8848 |
|  | NMI | 0.8530 | **0.8756** | 0.8622 | 0.8662 | 0.8620 |
| Plasschaert | ARI | 0.5946 | 0.5916 | 0.4754 | **0.6136** | 0.5194 |
|  | NMI | 0.6945 | **0.7116** | 0.6633 | 0.7040 | 0.6680 |
| Qx_Bladder | ARI | 0.7450 | **0.7579** | 0.7483 | 0.7460 | 0.7483 |
|  | NMI | 0.7934 | **0.8130** | 0.7979 | 0.7953 | 0.7979 |
| Qx_Limb_Muscle | ARI | 0.9918 | **0.9920** | 0.9918 | 0.9918 | 0.9918 |
|  | NMI | 0.9796 | **0.9803** | 0.9796 | 0.9796 | 0.9796 |
| Qx_Spleen | ARI | 0.9354 | **0.9371** | 0.8967 | 0.5678 | 0.9351 |
|  | NMI | 0.8658 | **0.8660** | 0.8494 | 0.7058 | 0.8649 |
| QS _Diaphragm | ARI | 0.9782 | **0.9893** | 0.9782 | 0.9782 | 0.9782 |
|  | NMI | 0.9632 | **0.9772** | 0.9632 | 0.9632 | 0.9632 |
| QS_Limb_Muscle | ARI | 0.9729 | 0.9444 | 0.9729 | **0.9733** | 0.9422 |
|  | NMI | 0.9544 | 0.9452 | 0.9544 | **0.9547** | 0.9423 |
| QS _Lung | ARI | 0.4905 | 0.4889 | 0.4902 | **0.4941** | 0.4921 |
|  | NMI | 0.7450 | 0.7403 | 0.7466 | **0.7470** | 0.7479 |
| Romanov | ARI | 0.6752 | **0.6946** | 0.6751 | 0.6757 | 0.6737 |
|  | NMI | 0.6802 | **0.7149** | 0.6754 | 0.6770 | 0.6759 |
| Tosches_turtle | ARI | 0.8160 | **0.8654** | 0.8152 | 0.8175 | 0.8447 |
|  | NMI | 0.8042 | 0.8076 | 0.8039 | 0.8067 | **0.8117** |
| Wang_lung | ARI | 0.9365 | **0.9396** | 0.9365 | 0.9365 | 0.9365 |
|  | NMI | 0.8651 | **0.8703** | 0.8651 | 0.8651 | 0.8651 |
| Young | ARI | 0.7727 | **0.7921** | 0.7127 | 0.7119 | 0.7574 |
|  | NMI | 0.8358 | **0.8477** | 0.8088 | 0.8073 | 0.8268 |
| **Average** | ARI | 0.7940 | 0.8169 | 0.7918 | 0.7736 | 0.7963 |
|  | NMI | 0.8244 | 0.8389 | 0.8243 | 0.8150 | 0.8241 |

**Table S2. Imputation results by scGCL with a different k value**

| **K_value** |  | **5** | **10** | **15** | **20** | **25** |
| --- | --- | --- | --- | --- | --- | --- |
| Adam | ARI | 0.8934 | 0.8655 | **0.9090** | 0.9035 | 0.8974 |
|  | NMI | 0.8879 | 0.8737 | **0.8944** | 0.8890 | 0.8838 |
| Alzheimer | ARI | 0.5861 | 0.5221 | **0.6197** | 0.5447 | 0.5412 |
|  | NMI | 0.6746 | 0.6525 | **0.7004** | 0.6608 | 0.6579 |
| Muraro | ARI | 0.8431 | 0.9056 | **0.9151** | 0.8843 | 0.9055 |
|  | NMI | 0.8533 | 0.8662 | **0.8742** | 0.8615 | 0.8664 |
| Plasschaert | ARI | 0.6036 | 0.5622 | 0.5875 | **0.6280** | 0.5794 |
|  | NMI | 0.6968 | 0.6898 | 0.7078 | **0.7100** | 0.6867 |
| Qx_Bladder | ARI | 0.7500 | 0.7500 | **0.7546** | 0.7500 | 0.7517 |
|  | NMI | 0.8002 | 0.8002 | **0.8085** | 0.8002 | 0.8024 |
| Qx_Limb_Muscle | ARI | 0.9918 | 0.9918 | **0.9920** | 0.9918 | 0.9918 |
|  | NMI | 0.9796 | 0.9796 | **0.9803** | 0.9796 | 0.9796 |
| Qx_Spleen | ARI | 0.9355 | 0.8580 | **0.9371** | 0.8580 | 0.5678 |
|  | NMI | 0.8656 | 0.8332 | **0.8662** | 0.8329 | 0.7058 |
| QS _Diaphragm | ARI | 0.9782 | 0.9782 | **0.9875** | 0.9782 | 0.9782 |
|  | NMI | 0.9632 | 0.9632 | **0.9727** | 0.9632 | 0.9632 |
| QS_Limb_Muscle | ARI | 0.9110 | 0.9729 | 0.9132 | 0.9729 | **0.9737** |
|  | NMI | 0.9293 | 0.9544 | 0.9325 | 0.9544 | **0.9546** |
| QS _Lung | ARI | 0.4936 | 0.5010 | **0.5064** | 0.4772 | 0.4734 |
|  | NMI | 0.7470 | 0.7486 | **0.7557** | 0.7354 | 0.7324 |
| Romanov | ARI | 0.6752 | 0.6756 | 0.6942 | 0.6756 | **0.6992** |
|  | NMI | 0.6753 | 0.6768 | **0.7154** | 0.6785 | 0.6957 |
| Tosches_turtle | ARI | 0.8160 | 0.8142 | **0.8427** | 0.8163 | 0.8051 |
|  | NMI | 0.8032 | 0.7997 | **0.8094** | 0.8052 | 0.7118 |
| Wang_lung | ARI | 0.9365 | 0.9365 | **0.9396** | 0.9365 | 0.9365 |
|  | NMI | 0.8651 | 0.8651 | **0.8703** | 0.8651 | 0.8651 |
| Young | ARI | 0.7876 | 0.7655 | **0.7920** | 0.7757 | 0.7804 |
|  | NMI | 0.8425 | 0.8312 | **0.8477** | 0.8359 | 0.8389 |
| **Average** | ARI | 0.8001 | 0.7928 | 0.8136 | 0.7995 | 0.7772 |
|  | NMI | 0.8274 | 0.8239 | 0.8383 | 0.8266 | 0.8103 |

**Table S3. Imputation results by scTAG with different distance measures**

| **Distance measures** |  | **cityblock** | **cosine** | **euclidean** | **L2** | **manhattan** |
| --- | --- | --- | --- | --- | --- | --- |
| Adam | ARI | 0.8687 | **0.8687** | 0.8441 | 0.8474 | 0.8593 |
|  | NMI | 0.8609 | **0.8609** | 0.8455 | 0.8473 | 0.8576 |
| Alzheimer | ARI | 0.5768 | 0.6647 | 0.5904 | 0.6510 | **0.6994** |
|  | NMI | 0.6259 | 0.6541 | 0.6281 | 0.6374 | **0.6607** |
| Muraro | ARI | 0.6468 | **0.8975** | 0.6506 | 0.5250 | 0.6041 |
|  | NMI | 0.7890 | **0.8581** | 0.7711 | 0.7088 | 0.7448 |
| Plasschaert | ARI | 0.5577 | **0.7398** | 0.5473 | 0.5573 | 0.5744 |
|  | NMI | 0.6899 | **0.7392** | 0.6690 | 0.6525 | 0.6948 |
| Qx_Bladder | ARI | 0.7511 | 0.4770 | **0.9975** | 0.6964 | 0.6691 |
|  | NMI | 0.7986 | 0.6169 | **0.9916** | 0.6975 | 0.6827 |
| Qx_Limb_Muscle | ARI | 0.8678 | 0.9471 | **0.9530** | 0.8920 | 0.8989 |
|  | NMI | 0.8811 | **0.9357** | 0.9336 | 0.8913 | 0.8937 |
| Qx_Spleen | ARI | 0.3285 | **0.6916** | 0.3118 | 0.1708 | 0.2412 |
|  | NMI | 0.4620 | **0.6145** | 0.4407 | 0.2611 | 0.3392 |
| QS _Diaphragm | ARI | 0.9505 | **0.9752** | 0.9206 | 0.9152 | 0.8808 |
|  | NMI | 0.9301 | **0.9501** | 0.8760 | 0.8730 | 0.8707 |
| QS_Limb_Muscle | ARI | **0.9736** | 0.9661 | 0.9639 | 0.9660 | 0.9708 |
|  | NMI | **0.9574** | 0.9416 | 0.9408 | 0.9429 | 0.9491 |
| QS _Lung | ARI | 0.6790 | 0.6147 | 0.6744 | 0.6634 | **0.6817** |
|  | NMI | 0.8015 | 0.7500 | 0.7998 | **0.8059** | 0.7925 |
| Romanov | ARI | 0.5682 | **0.6634** | 0.6524 | 0.6513 | 0.5826 |
|  | NMI | 0.6180 | **0.6895** | 0.6850 | 0.6842 | 0.6168 |
| Tosches_turtle | ARI | 0.8051 | 0.5852 | 0.7893 | **0.8201** | 0.8074 |
|  | NMI | 0.7118 | 0.7250 | 0.7158 | **0.7646** | 0.7375 |
| Wang_lung | ARI | **0.7757** | 0.7127 | 0.5601 | 0.6074 | 0.5754 |
|  | NMI | **0.6907** | 0.6380 | 0.5192 | 0.5533 | 0.5300 |
| Young | ARI | **0.6762** | 0.6488 | 0.6489 | 0.6448 | 0.6400 |
|  | NMI | **0.7703** | 0.7627 | 0.7414 | 0.7360 | 0.7509 |
| **Average** | ARI | 0.7161 | 0.7466 | 0.7217 | 0.6863 | 0.6918 |
|  | NMI | 0.7562 | 0.7669 | 0.7541 | 0.7183 | 0.7229 |

**Table S4. Imputation results by scTAG with a different k value**

| **K_value** |  | **5** | **10** | **15** | **20** | **25** |
| --- | --- | --- | --- | --- | --- | --- |
| Adam | ARI | 0.8331 | 0.8331 | **0.8687** | 0.8331 | 0.8441 |
|  | NMI | 0.8306 | 0.8306 | **0.8609** | 0.8306 | 0.8458 |
| Alzheimer | ARI | 0.5589 | 0.5609 | **0.6648** | 0.5868 | 0.5879 |
|  | NMI | 0.6313 | **0.6562** | 0.6542 | 0.6457 | 0.6375 |
| Muraro | ARI | 0.8508 | **0.8987** | 0.8975 | 0.6936 | 0.7070 |
|  | NMI | 0.8336 | **0.8672** | 0.8581 | 0.8215 | 0.8294 |
| Plasschaert | ARI | 0.6034 | 0.5490 | **0.7398** | 0.5763 | 0.5131 |
|  | NMI | 0.6536 | 0.6505 | **0.7392** | 0.6885 | 0.6583 |
| Qx_Bladder | ARI | 0.6952 | **0.9967** | 0.4770 | 0.5416 | 0.7558 |
|  | NMI | 0.7047 | **0.9887** | 0.6169 | 0.6499 | 0.7401 |
| Qx_Limb_Muscle | ARI | 0.8099 | 0.8680 | 0.9471 | **0.9817** | 0.8837 |
|  | NMI | 0.8548 | 0.8790 | 0.9357 | **0.9634** | 0.8965 |
| Qx_Spleen | ARI | 0.5549 | 0.3672 | **0.6916** | 0.4654 | 0.4266 |
|  | NMI | 0.5989 | 0.4913 | **0.6145** | 0.5715 | 0.5337 |
| QS _Diaphragm | ARI | **0.9807** | 0.9734 | 0.9752 | 0.9767 | 0.9522 |
|  | NMI | 0.9603 | **0.9606** | 0.9501 | 0.9586 | 0.9330 |
| QS_Limb_Muscle | ARI | 0.9738 | 0.9310 | 0.9661 | 0.9785 | **0.9811** |
|  | NMI | 0.9504 | 0.9026 | 0.9416 | 0.9551 | **0.9632** |
| QS _Lung | ARI | **0.6220** | 0.7053 | 0.6147 | 0.4565 | 0.4598 |
|  | NMI | 0.7757 | **0.7886** | 0.7500 | 0.7120 | 0.7069 |
| Romanov | ARI | **0.7531** | 0.7240 | 0.6634 | 0.6375 | 0.6566 |
|  | NMI | 0.6883 | **0.6921** | 0.6895 | 0.6638 | 0.6729 |
| Tosches_turtle | ARI | 0.8208 | 0.8026 | 0.5815 | **0.8296** | 0.8006 |
|  | NMI | **0.7676** | 0.7476 | 0.7250 | 0.7625 | 0.6973 |
| Wang_lung | ARI | 0.7217 | **0.7302** | 0.7127 | 0.5465 | 0.6312 |
|  | NMI | 0.6532 | **0.6532** | 0.6380 | 0.5098 | 0.5712 |
| Young | ARI | 0.4958 | 0.5193 | 0.6488 | 0.6715 | **0.6819** |
|  | NMI | 0.6708 | 0.6708 | 0.7627 | 0.7592 | **0.7767** |
| **Average** | ARI | 0.7339 | 0.7471 | 0.7464 | 0.6982 | 0.7058 |
|  | NMI | 0.7553 | 0.7699 | 0.7669 | 0.7494 | 0.7473 |
