# Supplementary material for: scGCL: an imputation method for scRNA-seq data based on graph contrastive learning
Source: Bioinformatics. 2023 Feb 24;39(3):btad098. doi: 10.1093/bioinformatics/btad098 (PMC9991516; doi:10.1093/bioinformatics/btad098)
Supplement: btad098_Supplementary_Data [file btad098_supplementary_data.zip › SupplementaryMaterials_S1_fig.docx]

# Supplementary Figures


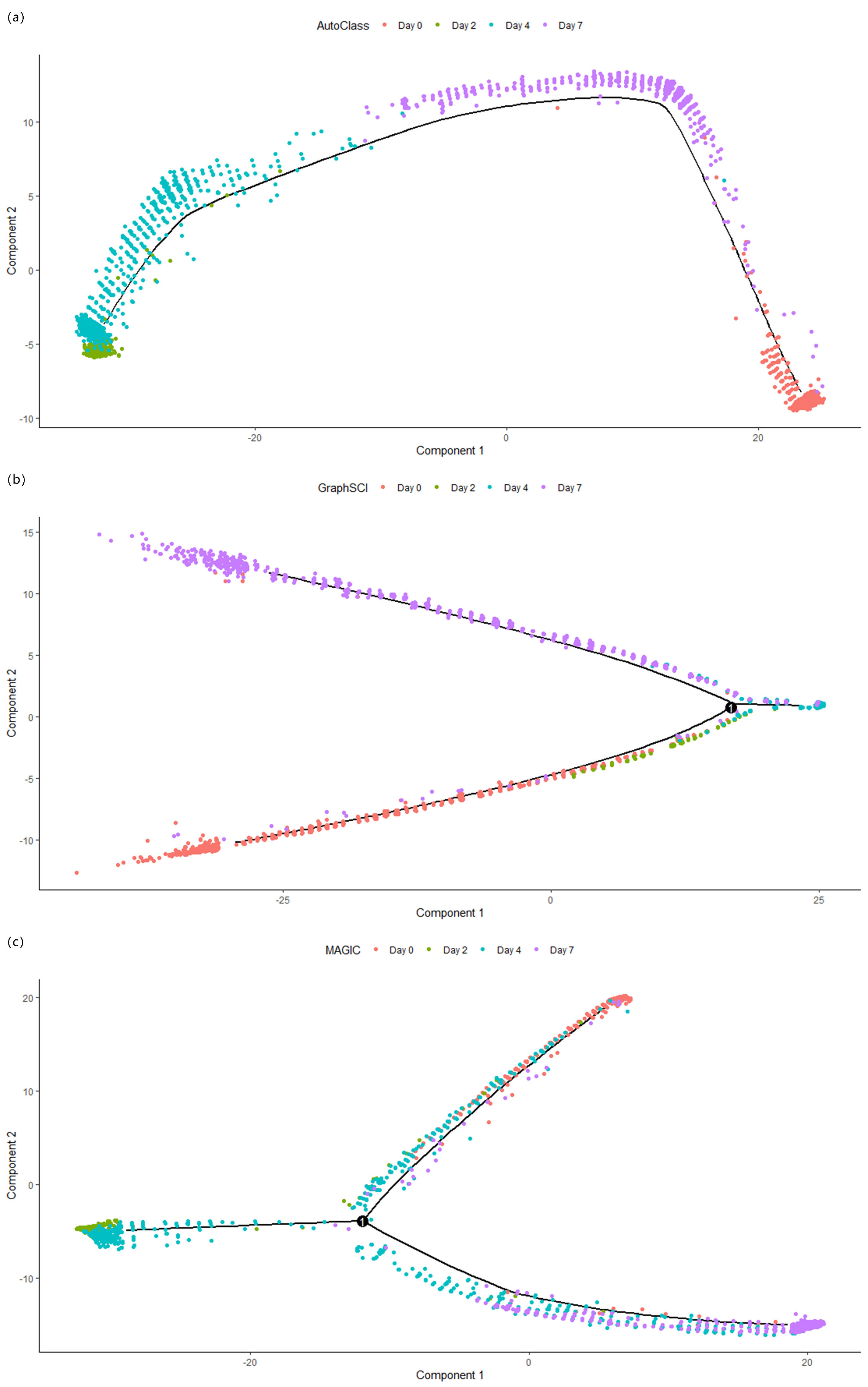
**Supplementary Figure 1：**Pseudotime analysis using the AutoClass-imputed matrix, GraphSCI-imputed matrix, and MAGIC-imputed matrix of the Klein data set via Monocle.


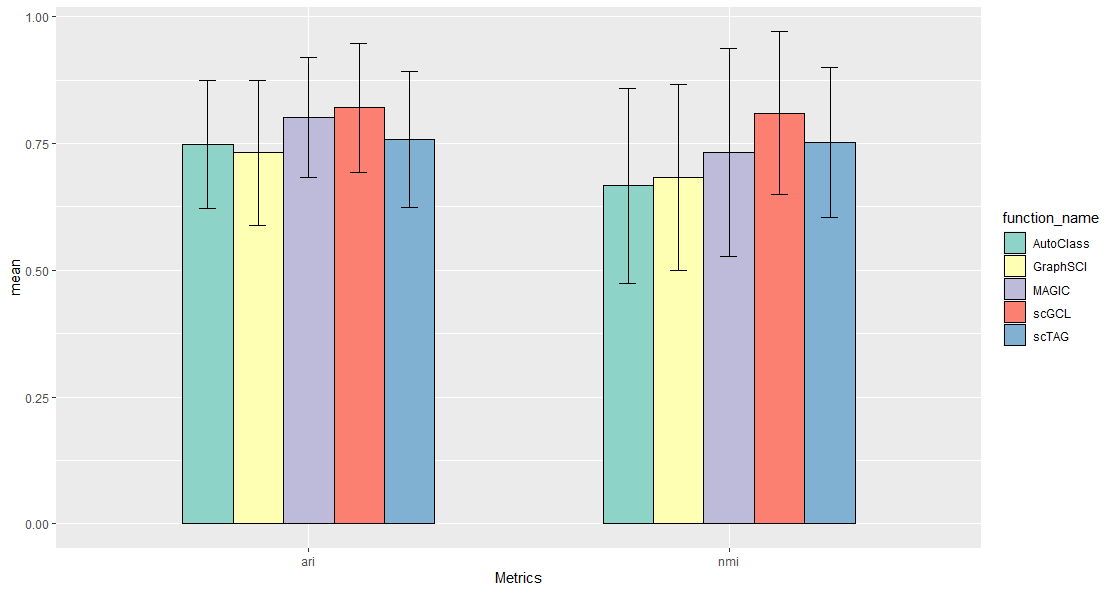


**Supplementary Figure 2：**Average ARI and NMI values (bar plot) and their variance (vertical lines)**.** scGCL outperforms other imputation and clustering methods.


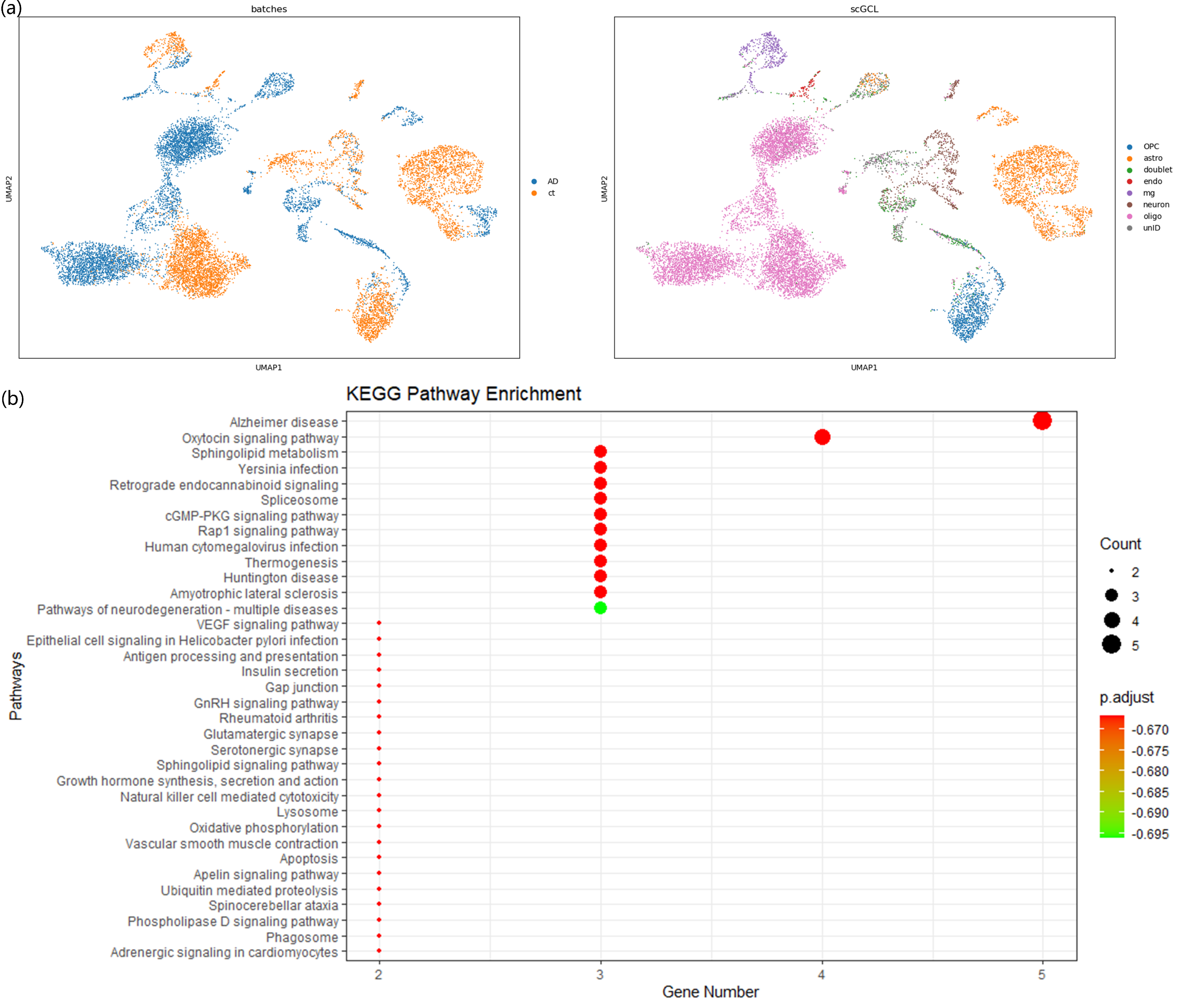


**Supplementary Figure 3：Clustering and UMAP visualization and KEGG pathway enrichment map of scGCL on Alzheimer dataset.** In the figure, **(a)** The UMAP map represents the visualization results of raw on Alzheimer dataset, **(b)** The KEGG pathway enrichment map by differentially expressed genes (DEG).


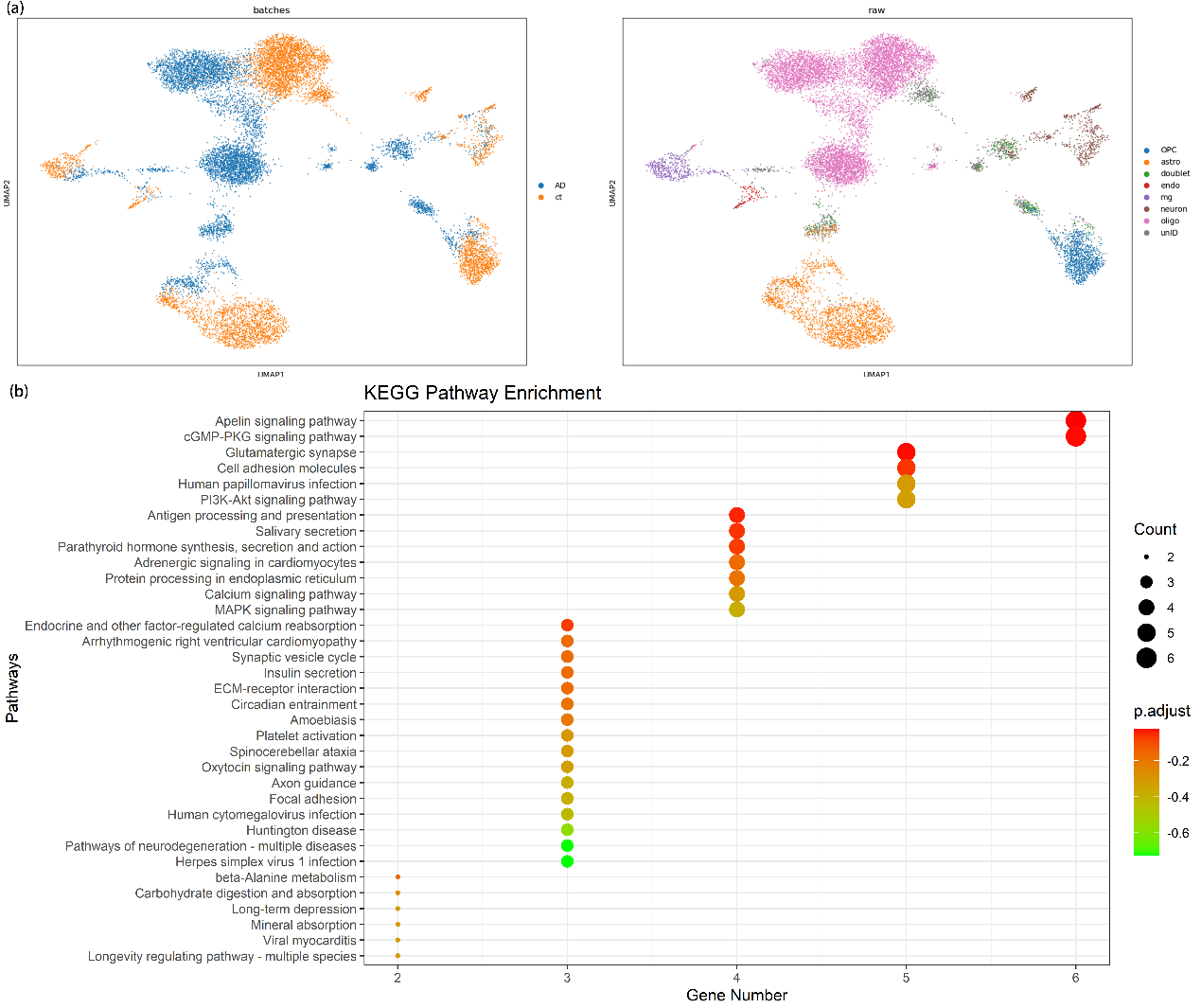


**Supplementary Figure 4：Clustering and UMAP visualization and KEGG pathway enrichment map of raw on Alzheimer dataset.** In the figure, **(a)** The UMAP map represents the visualization results of raw on Alzheimer dataset, **(b)** The KEGG pathway enrichment map by differentially expressed genes (DEG).


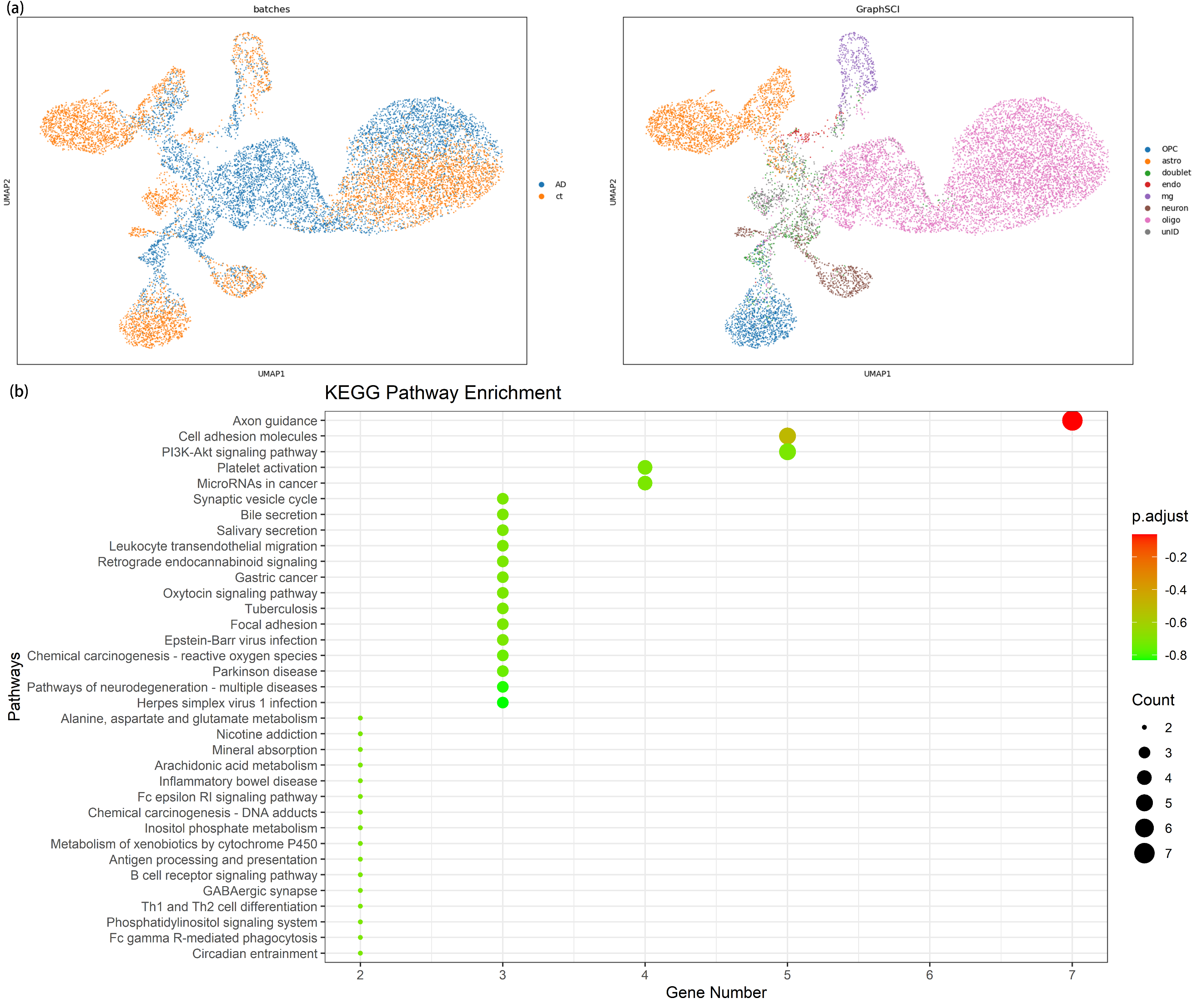


**Supplementary Figure 5：Clustering and UMAP visualization and KEGG pathway enrichment map of GraphSCI on Alzheimer dataset.** In the figure, **(a)** The UMAP map represents the visualization results of GraphSCI on Alzheimer dataset, **(b)** The KEGG pathway enrichment map by differentially expressed genes (DEG).


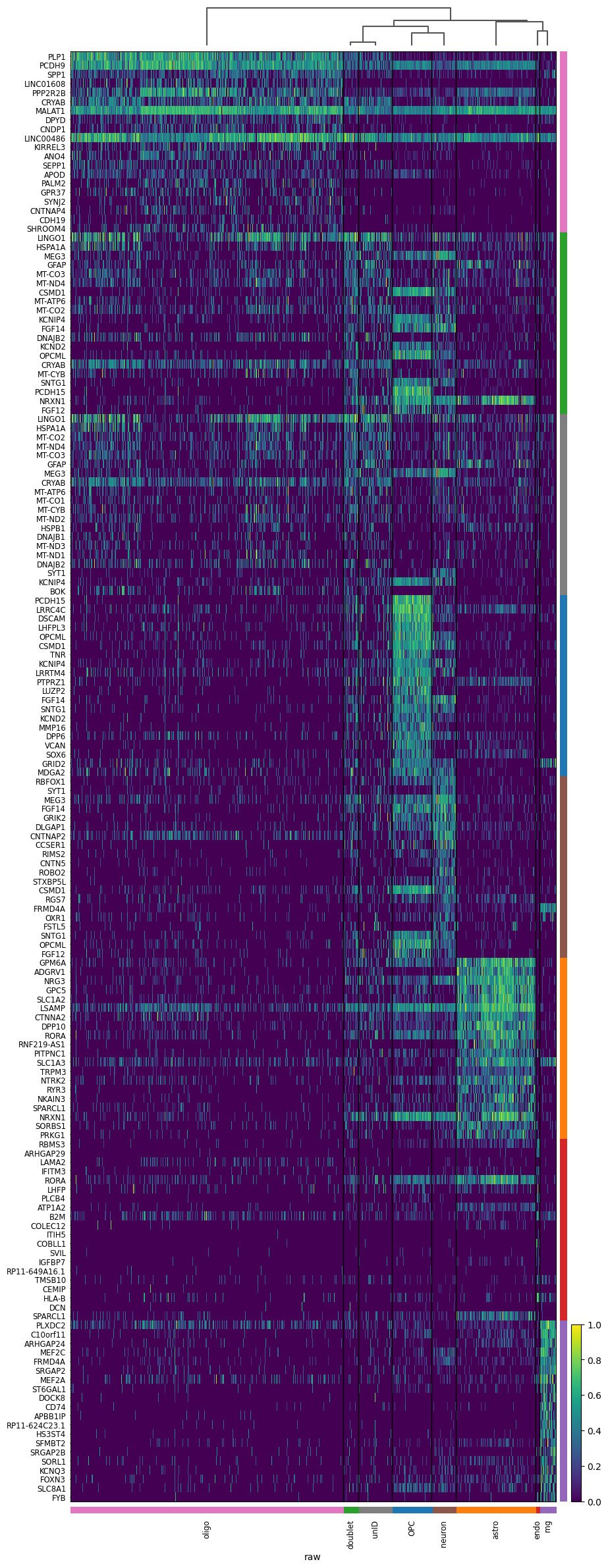


**Supplementary Figure 6：Expression of differentially expressed genes (DEG) generated by raw in different cell clusters.**


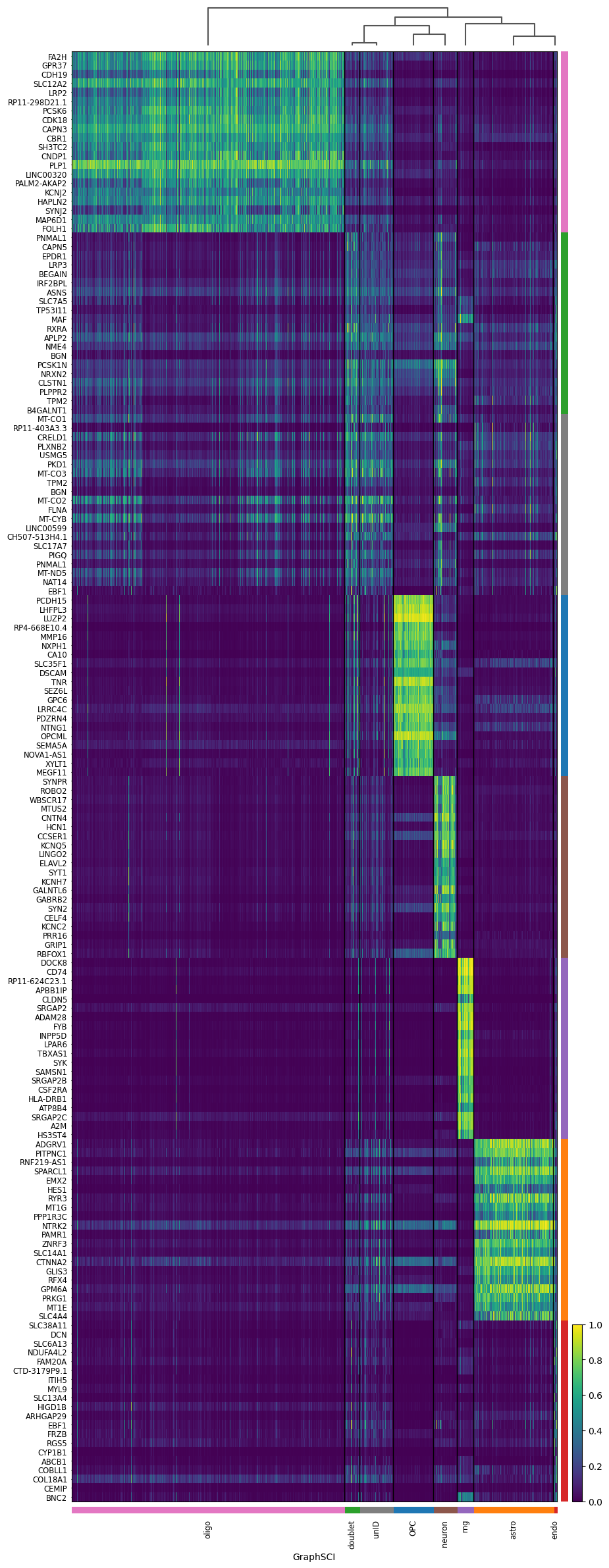


**Supplementary Figure 7：Expression of differentially expressed genes (DEG) generated by GraphSCI in different cell clusters.**


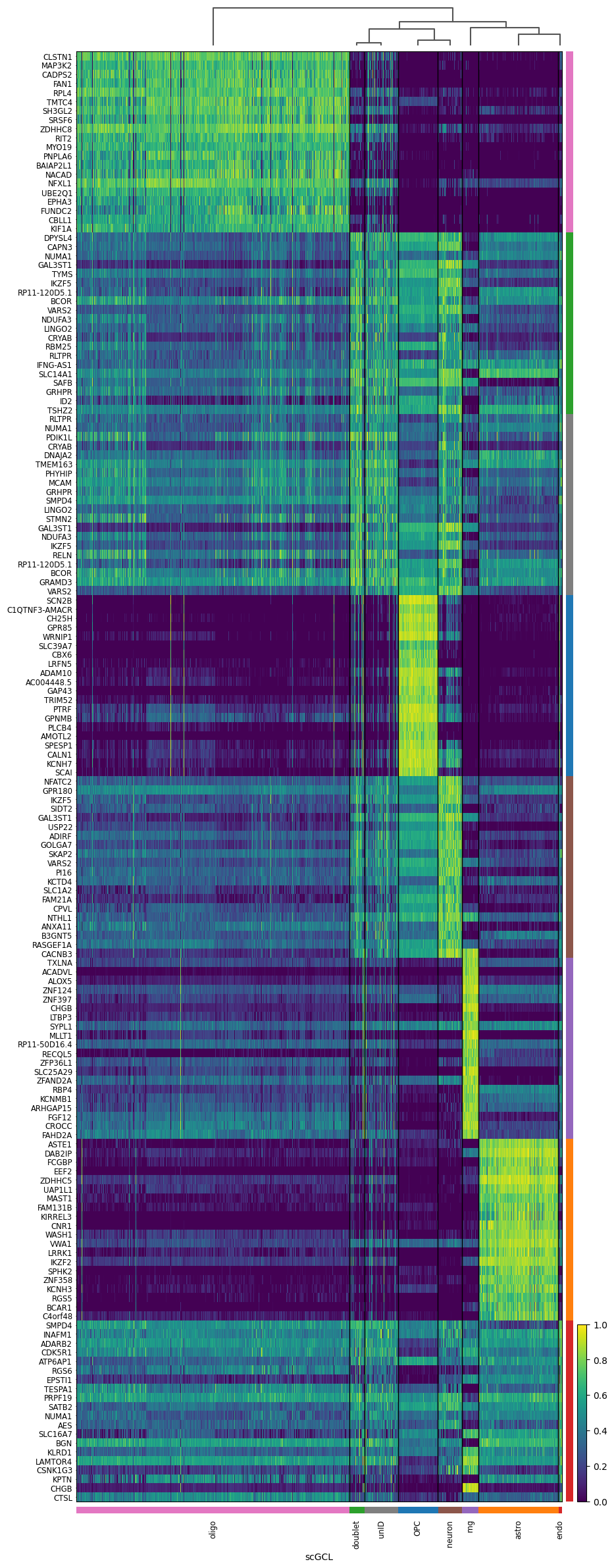


**Supplementary Figure 8：Expression of differentially expressed genes (DEG) generated by scGCL in different cell clusters.**


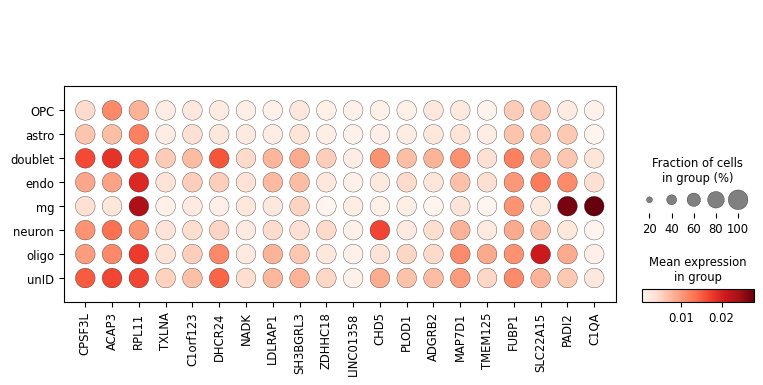


**Supplementary Figure 9：Expression of target genes generated by GraphSCI on Alzheimer dataset.**


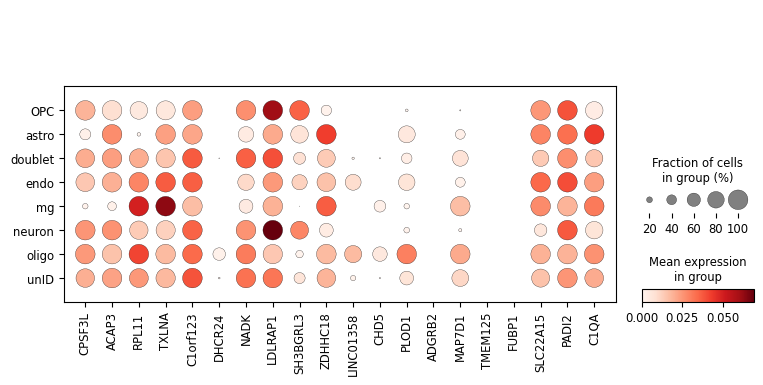


**Supplementary Figure 10：Expression of target genes generated by scGCL on Alzheimer dataset.**


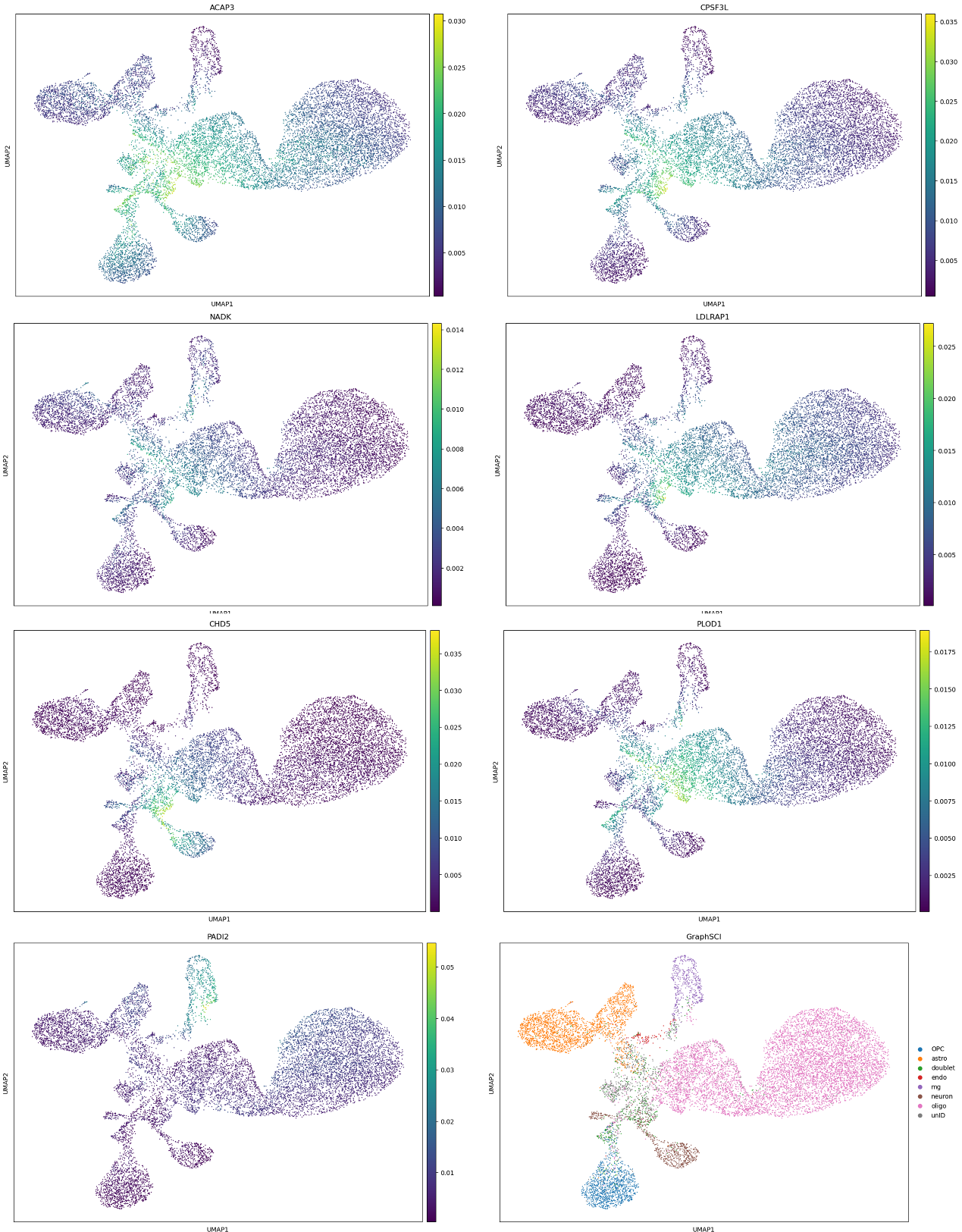


**Supplementary Figure 11：Distribution of target gene expression in different cell clusters by GraphSCI.**


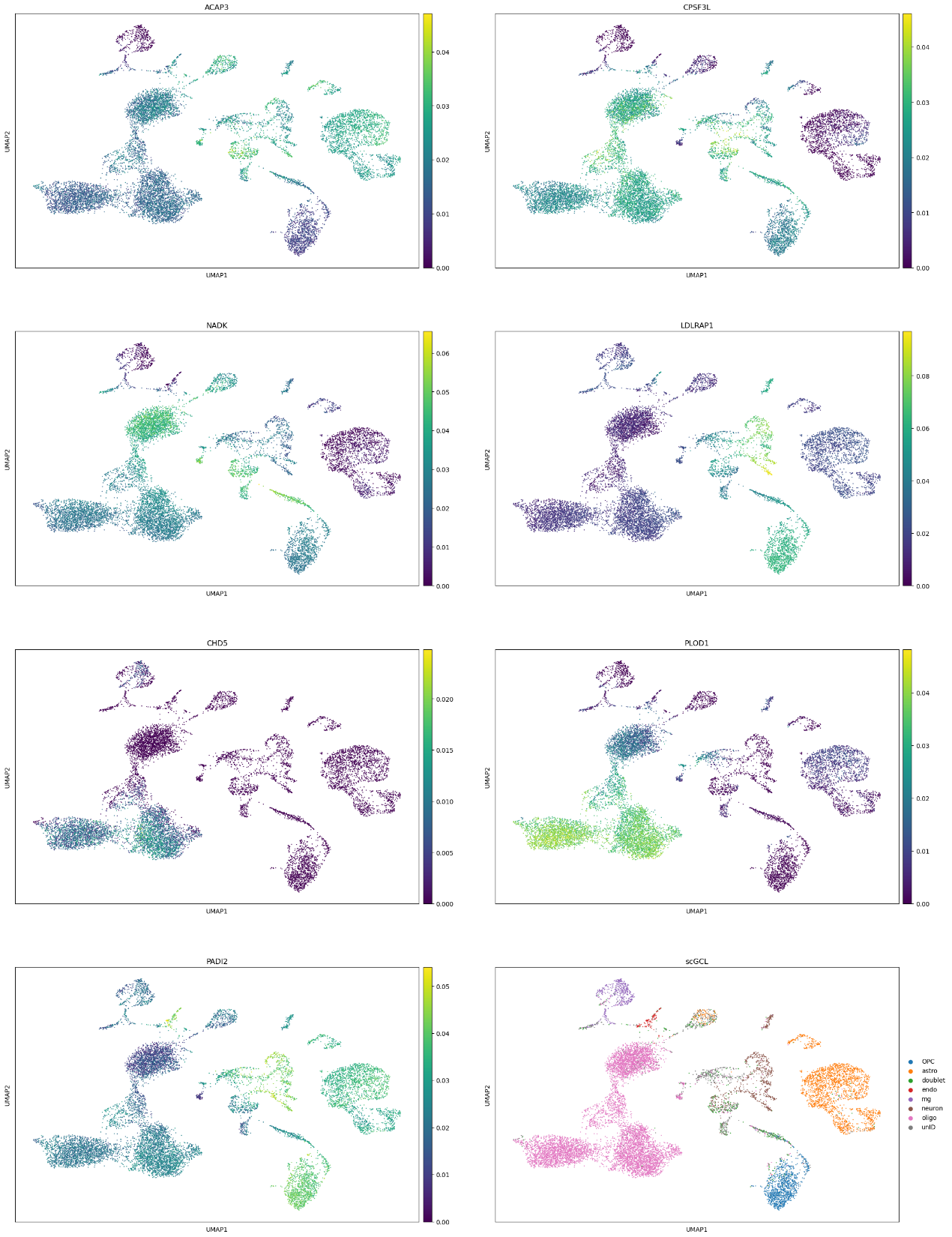


**Supplementary Figure 12：Distribution of target gene expression in different cell clusters by scGCL.**
